# Quantified density of performance-degrading near-interface traps in SiC MOSFETs

**DOI:** 10.1038/s41598-022-08014-5

**Published:** 2022-03-08

**Authors:** Mayank Chaturvedi, Sima Dimitrijev, Daniel Haasmann, Hamid Amini Moghadam, Peyush Pande, Utkarsh Jadli

**Affiliations:** 1 Queensland Micro- and Nanotechnology Centre, Griffith University, Brisbane, QLD 4111 Australia; 2 School of Engineering and Built Environment, Griffith University, Brisbane, QLD 4111 Australia; 3 Department of Electronics and Communication Engineering, Graphic Era (Deemed to be University), Dehradun, Uttarakhand 248002 India

**Keywords:** Electrical and electronic engineering, Characterization and analytical techniques, Characterization and analytical techniques

## Abstract

Characterization of near-interface traps (NITs) in commercial SiC metal–oxide–semiconductor field-effect transistors (MOSFETs) is essential because they adversely impact both performance and reliability by reducing the channel carrier mobility and causing threshold-voltage drift. In this work, we have applied a newly developed integrated-charge technique to measure the density of NITs that are active in the above-threshold region of commercial SiC MOSFETs. The results demonstrate that NITs trap about 10% of the channel electrons for longer than 500 ns.

## Introduction

SiC metal–oxide–semiconductor field-effect transistors (MOSFETs) are becoming a preferred choice for power switches used in a wide range of applications, such as high-frequency power converters, industrial motor drives, electric-vehicles, solar inverters, switch-mode power supplies, and power factor correction circuits. The advantage of SiC for power devices can be illustrated by Baliga’s figure of merit^1,2^,

1 $$\frac{{R}_{sp}}{{{V}_{B}}^{2}}=\frac{4}{{\varepsilon }_{s}{\mu }_{n}{{E}_{cr}}^{3}}$$

where ${R}_{sp}$ is the specific resistance of the drift region, ${V}_{B}$ is the breakdown voltage, ${\varepsilon }_{s}$ is the semiconductor permittivity, ${\mu }_{n}$ is the mobility of electrons in the drift region, and ${E}_{cr}$ is the critical electric field. The critical electric field of SiC is more than ten times higher than Si. Hence, for the same breakdown voltage, ${R}_{sp}$ for SiC becomes a thousand times lower than ${R}_{sp}$ of Si. Even though the substrate resistance of the SiC MOSFET is higher than in the Si MOSFET, the drift resistance is much smaller because the higher breakdown field of SiC enables a much thinner drift region^3^. Consequently, SiC MOSFETs were developed to offer lower on-resistance in comparison to Si MOSFETs and at higher blocking voltages^1–4^.

However, the advantages of SiC as a wide energy gap material have not been utilized fully because of interface and near-interface traps (NITs) in the gate dielectric^5^. A high density of fast band-edge traps exists in SiC MOS devices, which are active in the sub-threshold region^6^. For gate voltages higher than the threshold voltage $\left({V}_{T}\right)$, the Fermi level is in the conduction band due to the quantum-confinement effect^7,8^. Therefore, the electrons on interface and near-interface traps with energy levels aligned to the energy gap appear as fixed charge, increasing the threshold voltage. The threshold voltage is also impacted by NITs situated further away from the interface so that their response time is in the order of hours and days^9,10^. These traps are responsible for degraded reliability due to threshold-voltage drift. There are also fast near interface traps with energy levels aligned to the conduction band and around the Fermi level in the semiconductor. These traps continuously capture and release electrons, which reduces the average value of electron mobility in the MOSFET channel^11,12^. These traps, which degrade MOSFET performance, are the focus of this paper.

Generally, conductance and capacitance measurements are used to characterize interface traps and NITs. When these measurements are performed with above-threshold gate voltages, the conductance of SiC-based MOS capacitors tends to increase with frequency. An analogous behavior can be observed if the internal series resistance of the MOS capacitor is high. There exists a well-established method to compensate the impact of series resistance in capacitance measurements^13^. However, it was recently shown that the impact of NITs in SiC was misinterpreted as series resistance^8,14^. Several researchers have published results on the density and energy levels of NITs by utilizing MOS capacitors as test structures, as summarized and reviewed by Fiorenza et al*.*^15^ Pande et al.^16^, and Kimoto et al.^17^. That is mainly because companies do not provide the process specifications of commercial MOSFETs. However, the density of NITs in MOS capacitors can be different from the density of NITs in commercial MOSFETs due to different fabrication processes. Therefore, it is important to quantify the density of NITs with measurements performed on commercial MOSFETs.

Previously, numerous attempts have been made to detect traps in SiC MOSFETs aligned to the energy gap near the band edge. Saks et al*.* have profiled the density of interface traps near the band edges in MOSFETs by comparing theoretical *C–V* curves with measured *C–V* curves^18^. Few investigators have extracted interface-trap density based on subthreshold *I–V* characteristics^19–21^.

Potbhare et al*.* developed a physical model for the analysis of 4H-SiC MOSFETs. Interface trap densities were extracted by comparing simulated *I–V* curves with measured data in the subthreshold region, and the density of NITs was calculated from the difference between measured and simulated *I–V* characteristics in the above-threshold region^22^.

In this paper, we apply a newly developed integrated-charge technique^23^ to commercial SiC MOSFETs with the aim of quantifying—for the first time—the density of active NITs in the above-threshold region, which is not impacted by the inherent uncertainty of trap-free characteristics obtained by simulation. This is achieved by comparing measured values of integrated charge with response times ranging from 500 ns to 500 µs.

## Separating traps that impact performance from traps that impact reliability

**Figure 1:**
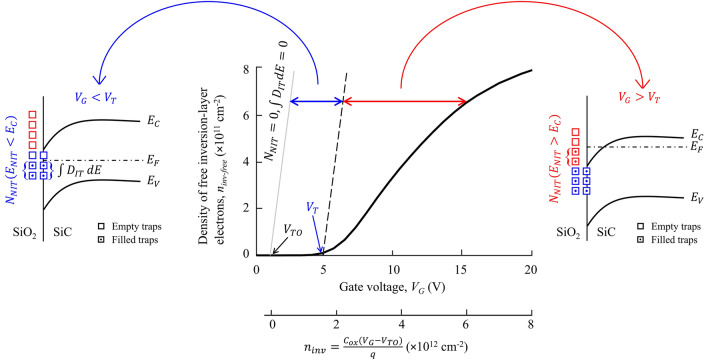
Illustration of the effects of interface- and near-interface traps with energy levels below the bottom of the conduction band (${E}_{NIT}<{E}_{C}$), shown with the blue symbols, and near-interface traps with energy levels above the bottom of the conduction band (${E}_{NIT}>{E}_{C}$), shown with the red symbols.

The active NITs in the above-threshold region, which are the focus of this paper, have energy levels aligned to the conduction band. It is important to distinguish these NITs from the interface traps and from NITs with energy levels aligned to the energy gap, which are commonly investigated by other authors^19–21^. Figure 1 illustrates that both the interface traps and near-interface traps with energy levels aligned to the energy gap (blue symbols) increase the threshold voltage, whereas the near-interface traps with energy levels aligned to the conduction band (red symbols) reduce the density of free electrons in the above-threshold region, which degrades the MOSFET performance due to a proportional reduction in the average channel-carrier mobility.

The density of electrons attracted to the SiC surface by the gate voltage $\left({V}_{G}\right)$ can be calculated using the charge sheet model^16^,

2 $${n}_{inv} = \frac{{C}_{ox}({V}_{G}-{V}_{TO})}{q}$$

where ${V}_{TO}$ is the trap-free threshold-voltage value, ${C}_{ox}$ is the gate-oxide capacitance per unit area, and $q$ is the electron charge. The density of inversion-layer electrons in the absence of carrier trapping is shown by the grey line in Fig. 1; this corresponds to the theoretical case of zero-density of NITs (${N}_{NIT}= 0$) and zero-density of interface traps per unit area ($\int {D}_{IT}dE=0$). However, in practical devices, the interface traps, and the NITs with energy levels below the conduction band $\left({E}_{NIT}<{E}_{C}\right)$ trap electrons attracted to the surface by the gate voltage and increase the threshold voltage from ${V}_{TO}$ to ${V}_{T}$. When NITs positioned further away from the interface capture electrons during MOSFET operation in electronic systems, the resulting threshold-voltage drift becomes a reliability issue.

In the above-threshold region ($${V}_{G}>{V}_{T}$$), the Fermi level crosses the bottom of the conduction band edge due to the quantum-confinement effect^7,8^. The NITs with energy levels above the bottom of the conduction band $$\left({E}_{NIT}>{E}_{C}\right)$$ become active as they capture and release electrons from the channel by tunneling^24^. The reduced average density of free inversion-layer electrons ($${n}_{inv-free})$$, by the factor $${n}_{inv-free}/{n}_{inv}$$, corresponds to a decreased channel current and, accordingly, to degraded performance of the MOSFET. As distinct from papers dealing with interface traps and near-interface traps impacting the threshold voltage, the aim of this paper is to quantify the NITs that are responsible for the current reduction and, hence, performance degradation in SiC MOSFETs.

## Applying the integrated-charge measurement technique to MOSFETs

To apply the integrated-charge measurement technique, the gate of the MOSFET under test was connected in series with an external resistor, ${R}_{EXT}$*,* whereas the source and the drain terminals were shorted and connected to ground. The measurements were performed by applying small voltage steps, $\Delta {V}_{step}$, to the series connection of the external resistor and gate capacitance, starting from the highest positive gate voltage and stepping it toward the lowest negative gate voltage (20 V to -20 V in this work). This is analogous to the standard quasi-static technique for capacitance measurement. However, the commercial instruments for quasi-static measurements require charge-integration times longer than tens of milliseconds and, because of that, the standard technique cannot detect charge trapping and release with time constants shorter than milliseconds^25,26^. We have recently-published an integrated-charge technique that can detect charge trapping and release times in the order of hundred nanoseconds^23^. In this paper, we applied this technique to detect and quantify for the first time the performance degrading near-interface traps in commercial MOSFETs. Utilizing a resistor connected in series with the gate oxide capacitance, a Tektronix DPO 7104 oscilloscope with Tektronix P6139B voltage probes was used to measure the voltage across the resistor and, therefore, to determine the current through the series connection of the resistor and gate capacitance. The measurements are not impacted by the MOSFET’s internal resistances because the current was obtained by the measurement of voltages across ${R}_{EXT}$. The measured current is integrated to obtain the charge in response to the applied voltage step, $q\Delta {N}_{carriers}$. This setup enabled measurements of charge in response to voltage steps as short as 500 ns. The capacitance is calculated from the fundamental relationship: $C = q\Delta {N}_{carriers}/\Delta {V}_{step}$.

The charge trapped for a longer time than the step interval, ${t}_{step}$, is not contributing to the current through the circuit. Consequently, the integrated charge ($q\Delta {N}_{carriers}$) obtained from measurements with shorter step intervals is smaller for the amount of charge trapped for longer than ${\tau }_{min}={t}_{step}$. This means that $\left[\Delta {N}_{carriers}\left(500 \mathrm{\mu s}\right)-\Delta {N}_{carriers}\left(500 \mathrm{ns}\right)\right]/{A}_{G}$, where ${A}_{G}$ is the gate area, is equal to the density of carriers per unit area captured by traps with response times longer than 500 ns and shorter than 500 µs. We label this density of trapped carriers by $\Delta {N}_{trapped}\left({\tau }_{min}=500 \mathrm{ns}\right)$. We selected 500 µs as the reference step interval because the density of traps with response times longer than 500 µs was too small to be detectable by this technique. To profile the density of traps with faster response times, we performed measurements with three step intervals, 500 ns, 5 µs, and 50 µs, and compared them with the reference $\Delta {N}_{carriers}$(500 µs).

## Measurement of reference Si MOSFET

**Figure 2:**
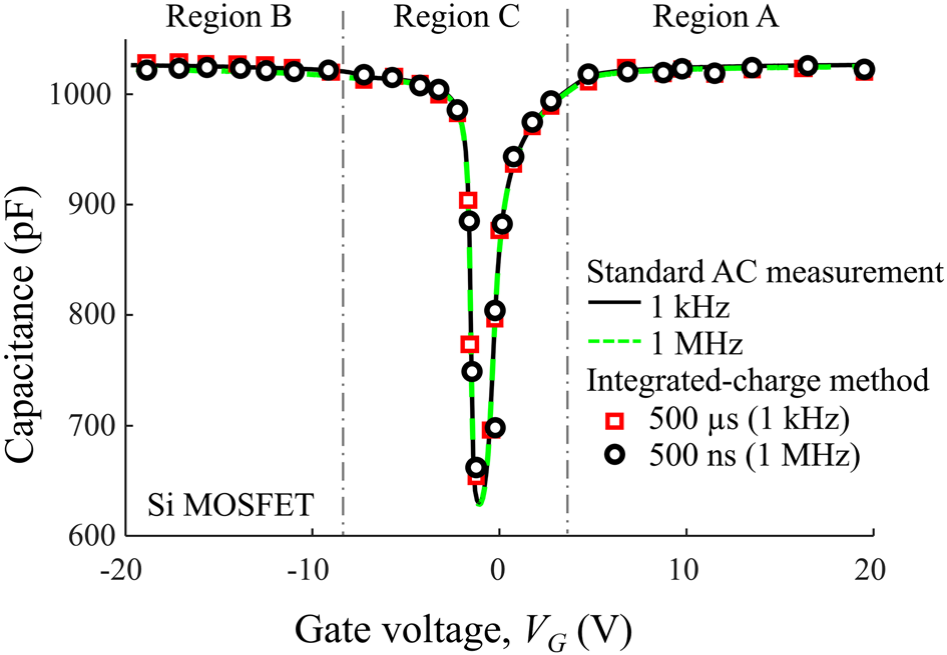
Capacitance–voltage curves for commercial N-channel Si power MOSFET.

Given that Si MOSFETs do not suffer from NITs^27^, we used a 100 V/9.7 A commercial Si MOSFET as a reference sample to verify the applicability of the integrated-charge technique. The on-resistance (${R}_{DS(on)}$) and gate-resistance (${R}_{G}$) of the Si MOSFET were 200 mΩ and 25 Ω respectively. The gate-to-source voltage (${V}_{GS}$) of the Si MOSFET was ± 20 V. Figure 2 shows the *C–V* curves obtained by the integrated-charge method in comparison with the standard AC measurements performed with an Agilent B1505A Power Device Analyzer. The agreement between these measurements verifies the applicability of the integrated-charge method and also demonstrates that there is no observable effect of carrier trapping.

**Figure 3:**
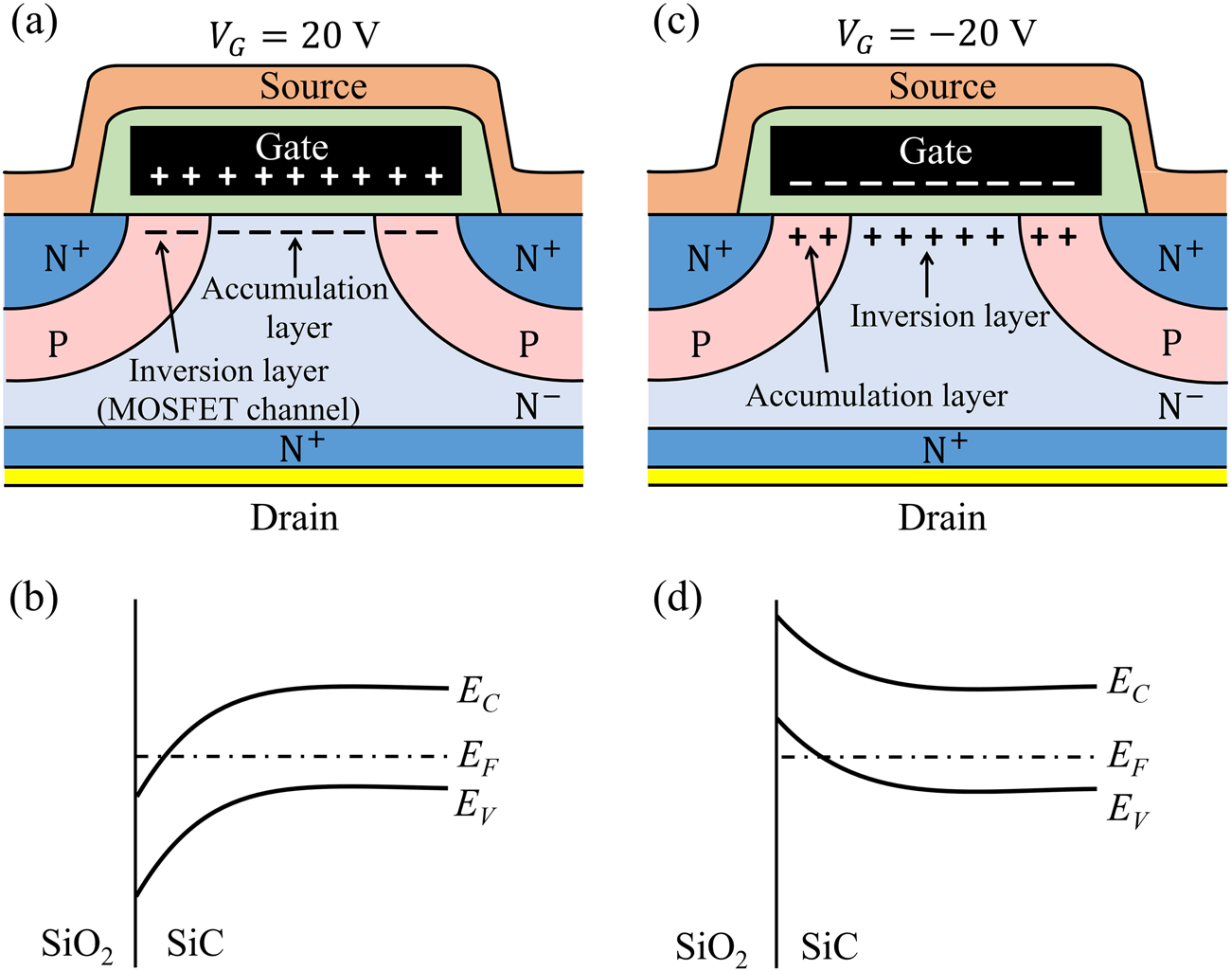
(**a**) Schematic cross-section of a planar power MOSFET, also called VDMOSFET, biased at${V}_{G}$= 20V; (**b**) Energy-band diagram showing that the Fermi level is inside the conduction band for region A; (**c**) Schematic cross-section of the planar power MOSFET biased at ${V}_{G}$= − 20V; (**d**) Energy-band diagram showing that the Fermi level is inside the valance band for region B.

The cross-section of a planar power MOSFET, shown in Fig. 3a, illustrates the capacitance at ${V}_{G}$ = 20 V. Here, the P-type body is inverted at the semiconductor surface, forming the MOSFET channel, whereas the drift region is accumulated. This means that the active area of the capacitor is equal to ${A}_{G}$ and the thickness of the capacitor is equal to the gate-oxide thickness (${t}_{ox}$). Therefore, the measured gate capacitance is equal to the gate oxide capacitance ${C}_{ox} = ({\varepsilon }_{ox}\times {A}_{G})/{t}_{ox}$ , where ${\varepsilon }_{ox}$ is permittivity of SiO_2_. Knowing the value of ${t}_{ox}$, the gate area can be obtained from the value of the measured capacitance, ${C}_{ox}$. If ${t}_{ox}$ is not known, it can be determined from Fowler–Nordheim tunneling by measuring ${I}_{G}-{V}_{G}$ characteristics^2^.

Note that the measured capacitance is equal to the gate-oxide capacitance in the entire region of gate voltages where the MOSFET channel is formed and the drift region is accumulated, which is labeled as Region A in Fig. 2. Due to the quantum-confinement effects, the Fermi level is inside the conduction band, as shown in Fig. 3b.

The cross-section of the MOSFET with ${V}_{G}$= − 20 V, shown in Fig. 3c, illustrates that the channel region of the MOSFET (the P-type region) is accumulated, and the surface of the drift region is inverted. Again, the measured capacitance is equal to ${C}_{ox}$ in this region of negative gate voltages, which is labeled as Region B in Fig. 2. In this case, the Fermi level is inside the valance band, as illustrated in Fig. 3d.

The reduced capacitance in Region C reflects the appearance of depletion layers in the P-type channel region and N-drift region, depending on the value of the gate voltage. In this region of gate-voltage values, the Fermi level is inside the energy gap.

## Measurement of SiC MOSFET

### *C–V* curves

**Figure 4:**
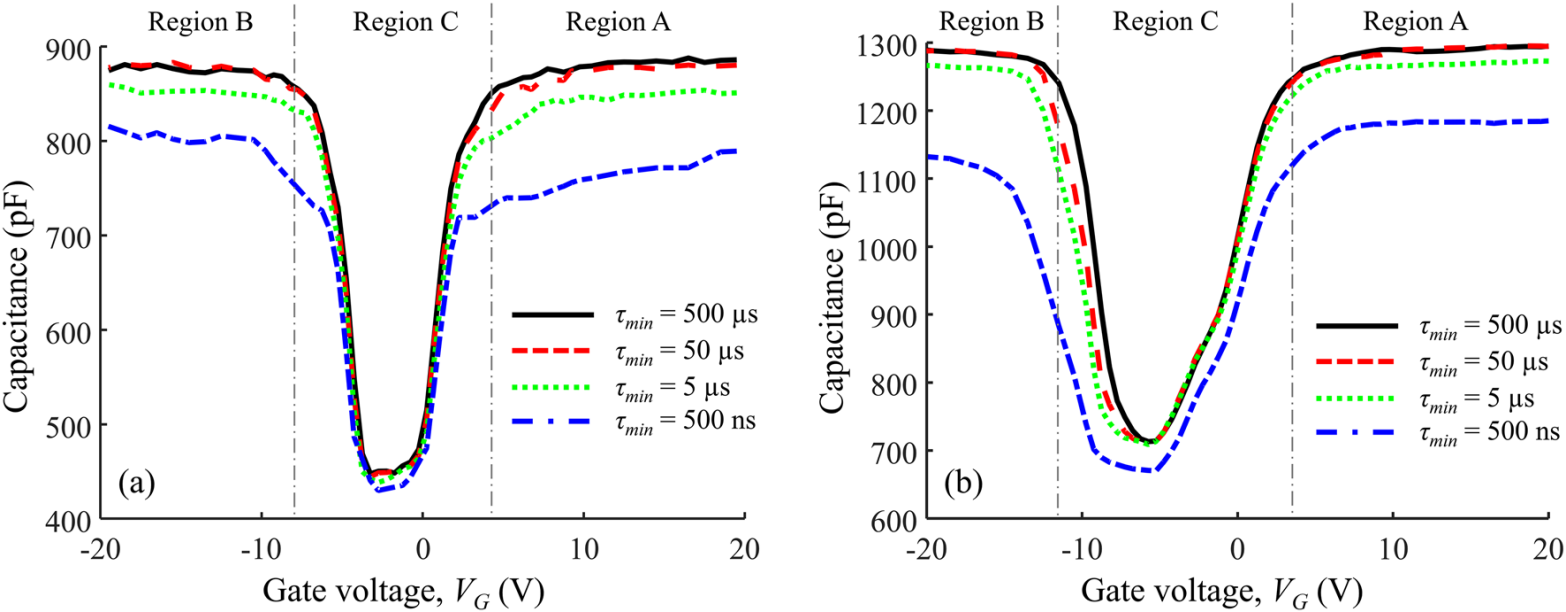
Capacitance–voltage curves of commercial N channel SiC Power MOSFETs for (**a**) manufacturer A, and (**b**) manufacturer B.

We performed measurements on commercial N-channel SiC Power MOSFETs from two different manufacturers, referred to as Manufacturer A and Manufacturer B. At least five devices of the same model have been tested and the results were repeatable. The commercial SiC MOSFETs have a standard gate-oxide thickness of 50 nm^28–30^ and the gate area was determined from the measured capacitance. The relevant device parameters are summarized in Table 1. Figure 4 shows the *C–V* curves obtained using the integrated-charge method for different response times.

**Table 1:** Device parameters of measured SiC MOSFETs.

Device Parameter	Manufacturer A	Manufacturer B
Drain-to-source voltage, ${V}_{DS}$ (V)	900	1200
Gate-to-source Voltage, ${V}_{GS}$ (V)	-4 to 15	-10 to 25
Continuous drain current, ${I}_{D}$ (A)	23	20
On-resistance, ${R}_{DS(on)}$(mΩ)	120	189
Gate area, ${A}_{G}$ (cm^-2^)	1.29 × 10^–2^	1.89 × 10^–2^
Internal gate resistance, ${R}_{G}$ (Ω)	13	7

Focusing on Region A (${V}_{G}>{V}_{T}$), it can be observed that the shorter response times correspond to reduced capacitance values. This apparent reduction corresponds to reduced charging/discharging of the capacitor due to the trapping of electrons with response times longer than the duration of the voltage step. More electrons remain trapped for the measurements with shorter voltage steps, which results in lower apparent capacitances. As illustrated by the red symbols in Fig. 1, the active NITs in this region are energetically located above the bottom of the conduction band, and are degrading the device performance by capturing electrons from the MOSFET channel. The operating gate voltage of the MOSFETs generally varies between 15 and 20 V, yet the existing techniques for characterization of near-interface traps are limited to sub-threshold gate voltages (${V}_{G}<{V}_{T}$) and traps with energy levels aligned to the energy gap (the blue symbols in Fig. 1).

Analogously to Region A, the reduction in capacitance for shorter times observed in Region B is due to trapping of holes. In Region C (${V}_{G}<{V}_{T}$), the energy levels of active traps are aligned to the energy gap, as illustrated by the blue symbols in Fig. 1. These NITs impact the reliability of the MOSFET due to threshold voltage shift. The results in Fig. 4 show that there is some capacitance reduction in Region C, but it corresponds to much lower level of trapping in comparison to Regions A and B. This is an important result obtained by the newly-developed integrated-charge method. It shows that the dominant impact is due to NITs with energy levels aligned to the conduction band (Region A) and the valence band (Region B), which cannot be detected by the standard characterization techniques that are focused on interface traps with energy levels within the energy gap (Region C)^6,31,32^.

### Density of trapped electrons

The total density of trapped charge with response times longer than ${\tau }_{min}$ at a given gate voltage ${V}_{G}$ can be calculated as:

3 $${N}_{Trapped}\left({\tau }_{min}\right)=\sum_{0}^{{V}_{G}}{\Delta N}_{trapped}\left({\tau }_{min}\right)$$

**Figure 5:**
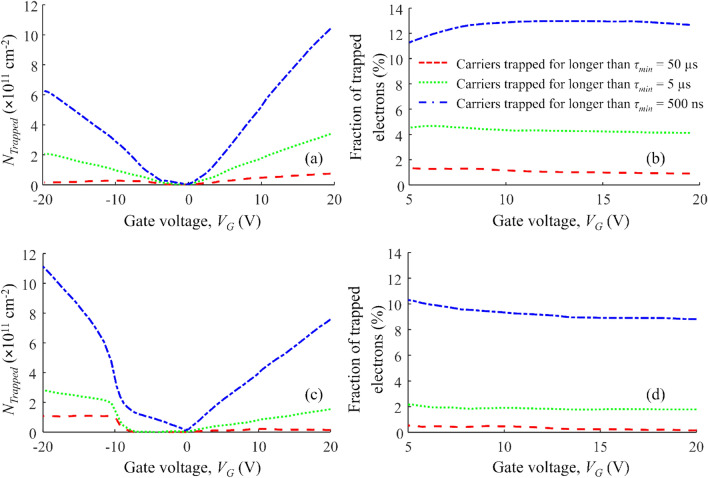
(**a**) The density of trapped charge with response times longer than ${\tau }_{min}$= 50 µs, 5 µs, and 500 ns for Manufacturer A; (**b**) the fraction of electrons trapped for longer than ${\tau }_{min}$, for Manufacturer A; (**c**) the density of trapped charge with response times longer than ${\tau }_{min}$= 50 µs, 5 µs, and 500 ns for Manufacturer B (**d**) the fraction of electrons trapped for longer than ${\tau }_{min}$ for Manufacturer B.

It is clear from the results shown in Fig. 5a, c that higher values of ${N}_{Trapped}$ were measured with shorter step intervals, corresponding to higher densities of NITs with shorter response times. Figure 5b, d shows the fraction of electrons that remain trapped for longer than ${\tau }_{min}$. These results show that the fraction of trapped electrons does not change significantly for gate voltages in the above-threshold region.

### Discussion

**Figure 6:**
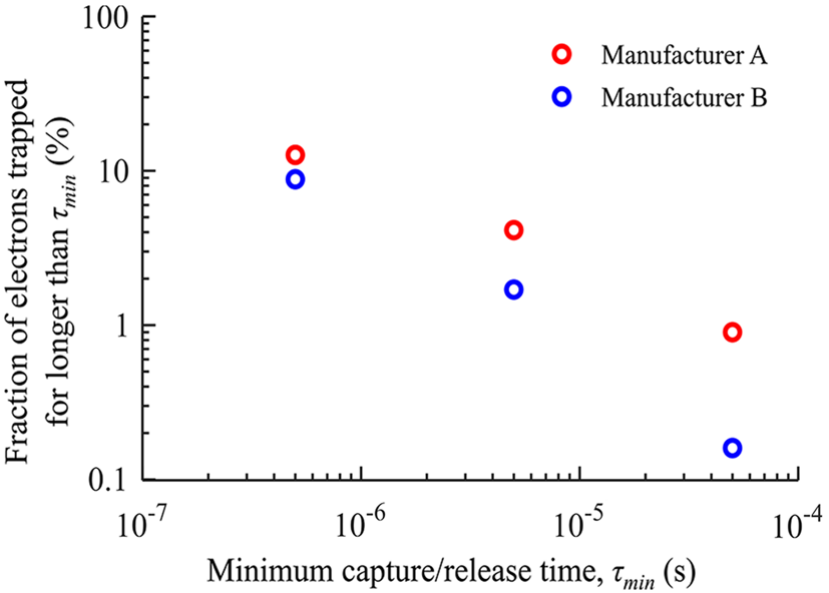
The fraction of electrons trapped for longer than ${\tau }_{min}$ at *V*_*G*_ = 20 V.

In Fig. 6, we show the fraction of channel electrons trapped for longer than ${\tau }_{min}$ at *V*_*G*_ = 20 V (the operating gate voltage). The density of trapped electrons with capture/release times longer than ${\tau }_{min}$ = 500 ns (the shortest step interval used in the measurements) is about 12% and 9% for Manufacturer A and Manufacturer B, respectively. However, the NITs closer to the SiC surface trap electrons for shorter times than 500 ns. Hence, the fraction of trapped channel electrons with shorter response times is higher.

These results are in agreement with the Hall effect measurements performed on 4H-SiC, which demonstrated that only about 20% of the electrons are not trapped at the highest gate voltages^33^. Another study based on Hall-effect measurements has also reported around 50% of trapped charge at the interface^34^.

## Conclusion

A recently-developed integrated-charge method was applied to quantify the density of NITs responsible for sub-optimal performance of commercial SiC MOSFETs. It was found that NITs with energy levels aligned to the conduction band trap about 10% of the channel electrons for longer than ${\tau }_{min}$= 500 ns, with estimated 80% of channel electrons trapped for longer than tens of nanoseconds. The presented data provide a unique profile of NITs for gate-voltage values above the threshold voltage. Device manufacturers can correlate this information to different steps and parameters in their fabrication processes, which can help them to improve the performance of their devices. On the other hand, this information will enable device users to compare and select MOSFETs from different manufacturers.

## Methods

**Figure 7:**
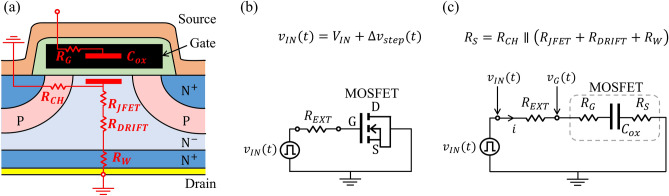
(**a**) Schematic cross-section of a planar power MOSFET biased in region A, showing the internal resistances and the gate-oxide capacitance, ${C}_{ox}$; (**b**) measurement circuit; (**c**) measurement circuit with equivalent circuit of the MOSFET.

Figure 7a shows the cross section of a planar power MOSFET with the internal resistances and the gate-oxide capacitance (${C}_{ox}$). The JFET region resistance (${R}_{JFET}$), drift region resistance (${R}_{DRIFT}$), and wafer resistance (${R}_{W}$) are connected in series, which are connected in parallel with the channel resistance (${R}_{CH}$). As shown in Fig. 7c, these resistances constitute the series resistance in the body of the MOSFET (${R}_{S}$). The internal gate-resistance of the MOSFET is shown by ${R}_{G}$.

The input voltage, ${v}_{IN}(t)$, comprising a DC-bias voltage (${V}_{IN}$) and a superimposed rectangular waveform ($\Delta {v}_{step}$) having the duty cycle of 50%, was applied to the $RC$ circuit as shown in Fig. 7b. The rectangular waveforms applied to the circuit were generated by a Tektronix AFG1022 function generator. The input voltage,${v}_{IN}(t)$, and the voltage between the gate and the grounded source of the MOSFET, ${v}_{G}(t)$, were measured by Tektronix DPO 7104 oscilloscope with Tektronix P6139B voltage probes as shown in Fig. 7c. The oscilloscope provides a clear trace over a single period by averaging thousands of cycles, which can be downloaded for further processing. It is also clear from Fig. 7c that the measurements are not impacted by MOSFET’s internal resistances because the current was obtained by measurement of voltages across ${R}_{EXT}$. This makes integrated-charge method immune to any variation in the internal resistance of the MOS structure. The carrier density in response to the rising edge of the applied pulse is obtained by integrating the current through ${R}_{EXT}$:

4 $${\Delta N}_{carriers}=\frac{1}{q{R}_{EXT}}\underset{0}{\overset{T/2}{\int }}\left({v}_{IN}-{v}_{G}\right)dt$$

where $T/2$ is half the period of the applied voltage pulses with the amplitude $\Delta {v}_{step}$. The values of ${\Delta N}_{carriers}$ are the same in response to the falling edge when the voltage pulse is at zero value, which can be obtained by integrating the current from $T/2$ to $T$.

Since trapped charge does not contribute to the current through the circuit, ${\Delta N}_{carriers}$ for shorter step intervals is smaller by the amount of charge trapped for longer than ${\tau }_{min}={t}_{step}$. Therefore, the following difference represents the density of trapped carriers per unit area:

5 $${\Delta N}_{trapped}=\frac{{\Delta N}_{carriers}({\tau }_{{min}_{slow}})-{\Delta N}_{carriers}({\tau }_{{min}_{fast}})}{{A}_{G}}$$

Square pulses of 1 kHz, 10 kHz, 100 kHz, and 1 MHz frequencies were used to profile NITs with response times shorter than 500 µs, 50 µs, 5 µs, and 500 ns, respectively. To ensure that the capacitor charges and then discharges within the half periods, the selection of suitable external series resistance are required. The criterion used for this selection in the time steps (${t}_{step}= T/2$) to be approximately equal to five time constants:

6 $${t}_{step} \approx 5\times \left({R}_{EXT}+{R}_{G}\right){C}_{ox}$$

This value of time step ensures that 99.3% of the charge with response times shorter than ${t}_{step}$ is detected. It should be noted that much smaller values of ${R}_{EXT}$, corresponding to much smaller time constants and ${t}_{step} \gg 5\times \left({R}_{EXT} + {R}_{G}\right){C}_{ox}$, should be avoided because the current drops to noise levels towards the end of ${t}_{step}$.

For the reference Si MOSFET series resistances of 24.82 kΩ and 17 Ω were used for the integrated-charge measurements with 500 µs and 500 ns, respectively. The series resistances used in the case of SiC MOSFETs are given in Table 2.

**Table 2:** External Series Resistance used for measurements of SiC MOSFETs.

**Frequency**	${{\varvec{t}}}_{{\varvec{s}}{\varvec{t}}{\varvec{e}}{\varvec{p}}}$	**External Series Resistance (**${{\varvec{R}}}_{{\varvec{E}}{\varvec{X}}{\varvec{T}}}$**)**	
		**Manufacturer A**	**Manufacturer B**
1 kHz	500 µs	80.3 kΩ	63.9 kΩ
10 kHz	50 µs	8 kΩ	6.5 kΩ
100 kHz	5 µs	832 Ω	619 Ω
1 MHz	500 ns	18 Ω	17.3 Ω

The measurements were performed for DC gate voltages from ${V}_{G}$ = 20 V to ${V}_{G}$ =  − 20 V. When measuring with ${V}_{G}$ from + 20 V to + 10 V and from − 10 V to − 20 V, the step size of the pulse was ${\Delta v}_{step}$ = 1 V. The voltage range from + 10 V to − 10 V was measured with the step size of ${\Delta v}_{step}$ = 500 mV. These step sizes were sufficiently small to ensure that the resulting changes in band bending and electric field are negligible.
